# Visualising and quantifying the usefulness of new predictors stratified by outcome class: The U-smile method

**DOI:** 10.1371/journal.pone.0303276

**Published:** 2024-05-20

**Authors:** Katarzyna B. Kubiak, Barbara Więckowska, Elżbieta Jodłowska-Siewert, Przemysław Guzik

**Affiliations:** 1 Department of Computer Science and Statistics, Poznan University of Medical Sciences, Poznan, Poland; 2 Division of Biostatistics, University of Minnesota, Minneapolis, Minnesota, United States of America; 3 Department of Cardiology - Intensive Therapy and Internal Medicine, Poznan University of Medical Sciences, Poznan, Poland; 4 University Centre for Sports and Medical Studies, Poznan University of Medical Sciences, Poznan, Poland; Jeonbuk Natiomal University, KOREA, REPUBLIC OF

## Abstract

Binary classification methods encompass various algorithms to categorize data points into two distinct classes. Binary prediction, in contrast, estimates the likelihood of a binary event occurring. We introduce a novel graphical and quantitative approach, the U-smile method, for assessing prediction improvement stratified by binary outcome class. The U-smile method utilizes a smile-like plot and novel coefficients to measure the relative and absolute change in prediction compared with the reference method. The likelihood-ratio test was used to assess the significance of the change in prediction. Logistic regression models using the Heart Disease dataset and generated random variables were employed to validate the U-smile method. The receiver operating characteristic (*ROC*) curve was used to compare the results of the U-smile method. The likelihood-ratio test demonstrated that the proposed coefficients consistently generated smile-shaped U-smile plots for the most informative predictors. The U-smile plot proved more effective than the *ROC* curve in comparing the effects of adding new predictors to the reference method. It effectively highlighted differences in model performance for both non-events and events. Visual analysis of the U-smile plots provided an immediate impression of the usefulness of different predictors at a glance. The U-smile method can guide the selection of the most valuable predictors. It can also be helpful in applications beyond prediction.

## Introduction

Binary classification methods are a variety of techniques and algorithms for the classification of data into two distinct classes or categories. If these techniques also determine the likelihood of belonging to a class, they are often called prediction methods. Some of these methods include Logistic Regression, Receiver Operating Characteristic (ROC) analysis, Matthews Correlation Coefficient, Support Vector Machines, Decision Trees and Neural Networks. These methods play a crucial role in both practical and scientific areas. For example, logistic regression models are used in economics to assess credit risk [1] and in marketing to target populations most likely to become customers for new products or services. The ROC curve, originally developed to detect enemy aircraft during World War II, has been used for decades in fields as diverse as psychology, medicine, radiology, biometrics, natural hazard forecasting, meteorology, and model performance evaluation [2–4]. It is also increasingly used in machine learning and data mining research. Although the Matthews correlation coefficient was originally introduced in biochemistry [5], it has gained popularity in several scientific disciplines, including software error prediction [6], pattern recognition [7], and medicine [8, 9]. In addition, binary classification problems are prevalent in social and data sciences, such as classifying social media users [10] and predicting mental health [11]. Accurate risk prediction models are crucial to making informed decisions in various areas, including medicine and epidemiology. Adding a new predictor to an existing model (a nested setting) may improve the model’s performance. New risk factors are constantly being discovered. It is essential to use appropriate statistical methods to assess their usefulness and ensure their practical applicability [12]. Some examples of this approach include the addition of high-density lipoprotein cholesterol, high-sensitivity C-reactive protein, or coronary artery calcium score to various risk assessment models [13–15].

Many methods assess the improvement in model performance offered by the new predictor [16–19]. The difference in the area under the receiver operating characteristic (*ROC*) curves (Δ*AUC*) of the models compared with and without the new predictor is a common approach. However, Δ*AUC* has several limitations. A useful predictor may increase the *AUC* too little to yield a significant difference, especially if the existing model already has a relatively high *AUC* [20–22]. Such an increase may be difficult to see when the two *ROC* curves are plotted on the same graph.

Another common limitation of the Δ*AUC* analysis and many standard methods is that they evaluate models globally, without examining the improvement in prediction separately for both outcome classes (e.g. healthy and diseased, or non-event and event groups).

However, some methods and measures used to assess the added value of new biomarkers or predictors in an existing model can be decomposed into separate components for the event and non-event classes. The Brier score (*BS*) and the net reclassification index (*NRI*) are common examples [23–26]. In this paper, we will abbreviate *NRI* to *I*.

The *BS* measures the accuracy of predicted probabilities for binary or categorical outcomes [26]. It reflects the magnitude of the error between the predicted probabilities and the actual outcomes. The *I* [25] is based on the number of prediction changes (called reclassifications) and does not quantify their magnitude. It compares the ability of two models to correctly classify individuals into risk categories based on their predicted probabilities and actual outcomes.

Although the *BS* and *I* remain popular methods, they have been criticised for many reasons. The *BS* can be sensitive to outliers and extreme predictions and may not reflect the clinical relevance of the predictions. On the other hand, the continuous *I* counts even minimal changes in the probability predicted by the models being compared. Furthermore, it does not consider the overall prediction accuracy. Unlike the Δ*AUC*, the *I* is not a proper scoring rule. [27–30].

In this methodological study, we propose and validate a novel U-smile method to evaluate the improvement in prediction due to the addition of a new marker to a set of reference markers to predict a binary outcome. The method includes the U-smile plot and new coefficients measuring the absolute (*BA*) and relative (*RB*) change in prediction compared with the reference method. As we will demonstrate later, our novel performance measures, *BA* and *RB*, are closely linked to the Brier score (*BS*). The *BA* is associated with the average change in prediction—the difference between *BS* values for the reference and new models. The *RB* can be directly expressed as the Brier skill score (*BSS*), which evaluates the relative change in prediction compared to the reference prediction. We also include the *I* coefficient for the U-smile method. Stratified by outcome class, the U-smile plot provides graphical information and the *BA*, *RB*, and *I* coefficients quantify the improvement in prediction. In this way, we show how easily the event and non-event classes can be further divided into subclasses of those whose prediction is improved or worsened by a new model. We further assessed the performance of the U-smile method using the likelihood-ratio test (LRT), and compared these results to those obtained with the DeLong’s test to evaluate Δ*AUC* for two correlated *ROC* curves.

## Methods

### Stratification of the prediction error

We consider a binary classification to predict the occurrence of a studied event, *D*, *D* ∈ {0, 1}. Let us consider two predictive models: a reference model based on a set of reference predictors, *X*, and a new model built by adding a candidate predictor, *Y*, to the reference model (nested models). We want to assess the degree of prediction improvement on the reference model offered by the new predictor. Equivalently, we want to determine how much of the prediction error of the reference model was reduced. For each individual, we examine model residuals, i.e. the differences between the observed outcome values and the predicted probabilities of the reference model (*δ*_*i*(*ref*)_) and the new model (*δ*_*i*_):

$$\begin{array}{cc} \delta_{i(ref)} & =d_{i}\left(1-p_{i(ref)}\right)+\left(1-d_{i}\right)p_{i(ref)}, \end{array}$$ (1)

$$\begin{array}{cc} \delta_{i} & =d_{i}\left(1-p_{i}\right)+\left(1-d_{i}\right)p_{i}, \end{array}$$ (2)

where, for each individual *i*, *i* = 1, …, *n*:

*p*_*i*(*ref*)_ and *p*_*i*_ are the predicted probabilities of the reference model and the new model, respectively;

*d*_*i*_ is the observed outcome value: *d*_*i*_ = 0 for individuals who do not develop the target event (the non-events) and *d*_*i*_ = 1 for those who develop this event (the events).

**Fig 1:**
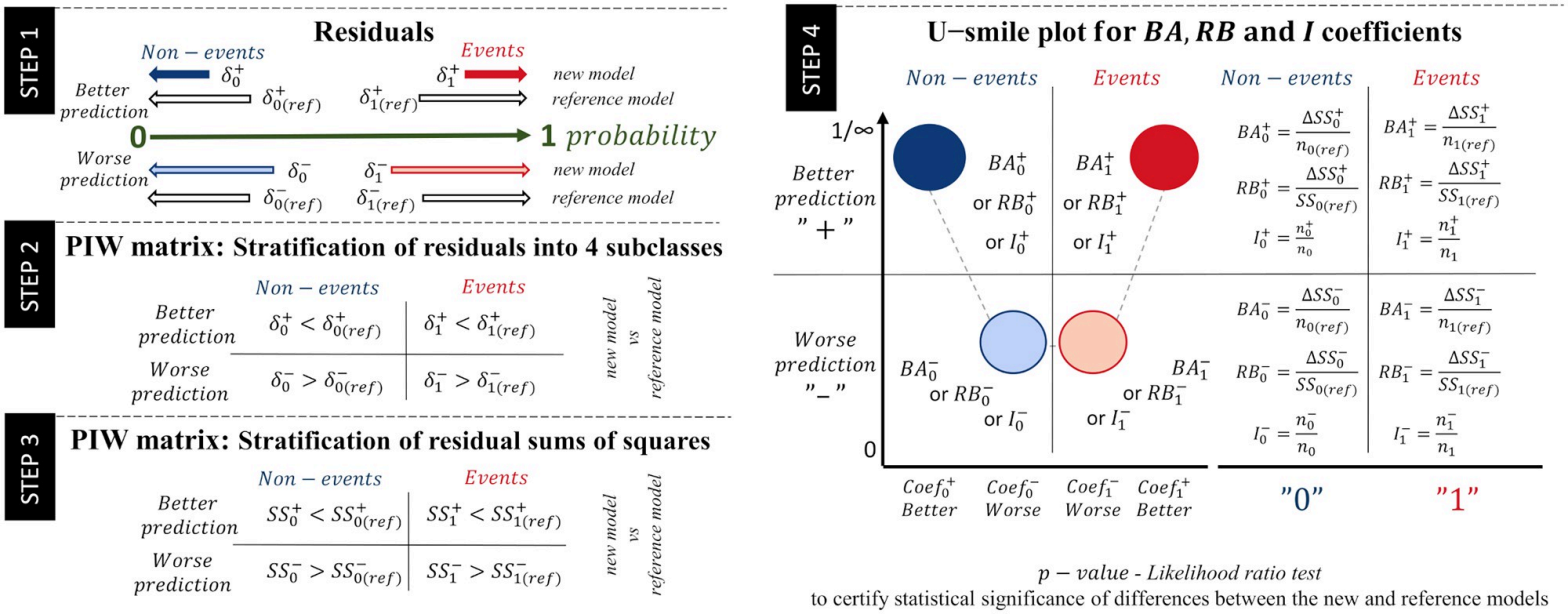
Steps to construct the *BA* and *RB* coefficients and the U-smile plot. Step 1: Shorten or lengthen the model residuals (*δ*). The reference model (subscript _(*ref*)_) includes the set of reference predictors, and a candidate predictor is added to the reference model to create a new model. The superscript ^+^ denotes better prediction, i.e. the new model shortens the residuals, and superscript ^−^ denotes worse prediction, i.e. the new model lengthens the residuals. Step 2: A prediction improvement-worsening (*PIW*) matrix is a formal cross-tabulation of individuals into four subclasses based on changes in the residual length of the new versus reference model. Step 3: Compared to each other, the residual sums of squares (*SS*) of the new model and the reference model in each of the four subclasses. Step 4: The U-smile plot. The *Y*-axis shows the coefficients labelled Coeff—a general abbreviation which, depending on the type of coefficient presented, may be replaced by *BA* (the size of the absolute average change in residuals), *RB* (the size of the relative change in residuals), or *I* (the proportion of individuals with residuals change). The *X*-axis shows the division into four subclasses: $Coef_{0}^{+}$ means better prediction for the non-events (dark blue circle), $Coef_{0}^{-}$—worse prediction for the non-events (light blue circle), $Coef_{1}^{-}$—worse prediction for the events (light red circle), and $Coef_{1}^{+}$– better prediction for the events (dark red circle). The connected cilcles form a smile when the magnitude of the prediction improvement (the external dark blue and red cilcles in the plot) is greater than that of the prediction worsening (the inner light blue and red cilcles in the plot).

The smaller the model residuals, the more accurate the predictions; the greater the model residuals, the more missed the predictions. If the new predictor improves on the reference model, then the residuals of the new model will be shorter than those of the reference model (Fig 1 Step 1).

Each individual is cross-tabulated by outcome class (non-event or event) and prediction subclass (improvement or worsening). By comparing the size of the residuals, we obtain a four-subclass prediction improvement-worsening (*PIW*) matrix. (Fig 1 Step 2):

$$\begin{array}{c} \delta_{0}^{+}<\delta_{0(ref)}^{+}, \end{array}$$ (3)

$$\begin{array}{c} \delta_{0}^{-}>\delta_{0(ref)}^{-}, \end{array}$$ (4)

$$\begin{array}{c} \delta_{1}^{-}>\delta_{1(ref)}^{-}, \end{array}$$ (5)

$$\begin{array}{c} \delta_{1}^{+}<\delta_{1(ref)}^{+}, \end{array}$$ (6)

where subscripts _0_ and _1_ denote the non-events and events, respectively, and superscripts ^+^ and ^−^ denote the better and worse prediction, respectively. This notation will indicate the stratification by outcome class and subclass.

### Stratification of the residual sums of squares

Let $SS_{\left(ref\right)}=\sum_{i=1}^{n}\delta_{i(ref)}^{2}$ be the residual sum of squares of the reference model. By stratifying *SS*_(*ref*)_ by outcome class and subclass (Fig 1 Step 3), we have:

$$\begin{array}{c} SS_{\left(ref\right)}=SS_{0(ref)}+SS_{1(ref)}=SS_{0(ref)}^{+}+SS_{0(ref)}^{-}+SS_{1(ref)}^{-}+SS_{1(ref)}^{+}. \end{array}$$ (7)

We consider the squared residuals to be the model prediction error. Therefore, the overall prediction error of the reference model is *SS*_(*ref*)_, and it was decomposed into *SS*_0(*ref*)_ and *SS*_1(*ref*)_, and further into $SS_{0(ref)}^{+}$, $SS_{0(ref)}^{-}$, $SS_{1(ref)}^{-}$, and $SS_{1(ref)}^{+}$.

Analogously, let $SS=\sum_{i=1}^{n}\delta_{i}^{2}$ be the residual sum of squares of the new model. By stratifying *SS* by outcome class and subclass, we have:

$$\begin{array}{c} SS=SS_{0}+SS_{1}=SS_{0}^{+}+SS_{0}^{-}+SS_{1}^{-}+SS_{1}^{+}. \end{array}$$ (8)

*SS* is the overall prediction error of the new model, *SS*_0_ and *SS*_1_ are the prediction error remaining within the non-event and event classes, respectively, and $SS_{0}^{+}$, $SS_{0}^{-}$, $SS_{1}^{-}$, $SS_{1}^{+}$ are the prediction error remaining within each subclass.

Stratifying Δ*SS* by outcome subclass, let us define the difference between the prediction error of the reference model and the new one:

$$\begin{array}{c} \Delta SS=SS_{\left(ref\right)}-SS=\Delta SS_{0}^{+}-\Delta SS_{0}^{-}+\Delta SS_{1}^{+}-\Delta SS_{1}^{-}, \end{array}$$ (9)

where:

$$\begin{array}{c} \Delta SS_{0}^{+}=SS_{0(ref)}^{+}-SS_{0}^{+}, \end{array}$$ (10)

$$\begin{array}{c} \Delta SS_{0}^{-}=SS_{0}^{-}-SS_{0(ref)}^{-}, \end{array}$$ (11)

$$\begin{array}{c} \Delta SS_{1}^{-}=SS_{1}^{-}-SS_{1(ref)}^{-}, \end{array}$$ (12)

$$\begin{array}{c} \Delta SS_{1}^{+}=SS_{1(ref)}^{+}-SS_{1}^{+}. \end{array}$$ (13)

$\Delta SS_{0}^{+}$ and $\Delta SS_{1}^{+}$ express the size of the prediction improvement, while $\Delta SS_{0}^{-}$ and $\Delta SS_{1}^{-}$ express the size of the prediction worsening in the corresponding outcome class.

### The *BA* and *RB* coefficients

We define two coefficients, *BA* and *RB*. Both describe the change in prediction error (Δ*SS*) and theoretically behave similarly only for balanced data where *n*_0_ ≈ *n*_1_ and *SS*_0(*ref*)_ ≈ *SS*_1(*ref*)_. However, their interpretation is different. The *BA* coefficients refer to the average absolute change in prediction between a new and a reference model. The *RB* coefficients refer to the change relative to the prediction of the reference model. For a detailed definition and description of the coefficients, we refer to the subsections BA and RB. A synthetic description of the interpretation and the coefficients range can be found in Table 1.

**Table 1:** Range and interpretation of the *BA*, *RB*, and *I* coefficients in the subclasses and the net coefficients for the classes.

Breakdown of individuals	absolute average change	relative change	proportion of individuals with changed prediction
**The non-event class**	*BA*_0_ ∈ [−1, 1]	*RB*_0_ ∈ (−∞, 1]	*I*_0_ ∈ [−1, 1]
**The non-event subclasses:**			
with better prediction	$BA_{0}^{+}\in \left[0,1\right]$	$RB_{0}^{+}\in \left[0,1\right]$	$I_{0}^{+}\in \left[0,1\right]$
with worse prediction	$BA_{0}^{-}\in \left[0,1\right]$	$RB_{0}^{-}\in \left[0,\infty \right)$	$I_{0}^{-}\in \left[0,1\right]$
**The event class**	*BA*_1_ ∈ [−1, 1]	*RB*_1_ ∈ (−∞, 1]	*I*_1_ ∈ [−1, 1]
**The event subclasses:**			
with worse prediction	$BA_{1}^{-}\in \left[0,1\right]$	$RB_{0}^{-}\in \left[0,\infty \right)$	$I_{0}^{-}\in \left[0,1\right]$
with better prediction	$BA_{1}^{+}\in \left[0,1\right]$	$RB_{0}^{+}\in \left[0,1\right]$	$I_{0}^{+}\in \left[0,1\right]$
**Interpretation of the range of coefficients**			
For classes:			
negative values = worse prediction of the new model,			
positive values = better prediction of the new model.			
For subclasses with better prediction of the new model:			
lower limit = no improvement, upper limit = maximum improvement.			
For subclasses with worse prediction of the new model:			
lower limit = no worsening, upper limit = maximum worsening.			

#### The *BA* coefficients

We define a family of the *BA* coefficients (Fig 1 Step 4), stratified by outcome subclass as:

$$\begin{array}{cc} BA_{0}^{+} & =\frac{\Delta SS_{0}^{+}}{n_{0}}, \end{array}$$ (14)

$$\begin{array}{cc} BA_{0}^{-} & =\frac{\Delta SS_{0}^{-}}{n_{0}}, \end{array}$$ (15)

$$\begin{array}{cc} BA_{1}^{-} & =\frac{\Delta SS_{1}^{-}}{n_{1}}, \end{array}$$ (16)

$$\begin{array}{cc} BA_{1}^{+} & =\frac{\Delta SS_{1}^{+}}{n_{1}}, \end{array}$$ (17)

where *n*_0_ and *n*_1_ are the numbers of non-events and events, respectively (*n*_0_ + *n*_1_ = *n*).

The *BA* coefficients quantify absolute average changes in the prediction of the reference model: $BA_{0}^{+}$ and $BA_{1}^{+}$ express an absolute average improvement of the prediction, while $BA_{0}^{-}$ and $BA_{1}^{-}$ express an absolute average worsening of the prediction in the corresponding outcome class.

The range of $BA_{0}^{+}$, $BA_{0}^{-}$, $BA_{1}^{-}$ and $BA_{1}^{+}$ is [0, 1]. Values closer to 1 indicate greater differences between the reference and new predictions in the corresponding outcome subclass. Therefore, higher values of $BA_{0}^{+}$ and $BA_{1}^{+}$ support adding the new predictor to the reference model, while higher values of $BA_{0}^{-}$ and $BA_{1}^{-}$ favour the reference model.

We also define the net *BA* coefficients stratified by outcome class as:

$$\begin{array}{cc} BA_{0} & =BA_{0}^{+}-BA_{0}^{-}, \end{array}$$ (18)

$$\begin{array}{cc} BA_{1} & =BA_{1}^{+}-BA_{1}^{-}. \end{array}$$ (19)

The range of *BA*_0_ and *BA*_1_ is [−1, 1]. Positive values mean better prediction, negative values mean worse prediction, while 0 equals no net improvement of the new predictor. The *BA*_0_ and *BA*_1_ coefficients express the net absolute average improvement in prediction for the non-events and events, respectively.

#### The *RB* coefficients

We define a family of the *RB* coefficients (Fig 1 Step 4), stratified by outcome subclass as:

$$\begin{array}{cc} RB_{0}^{+} & =\frac{\Delta SS_{0}^{+}}{SS_{0(ref)}}, \end{array}$$ (20)

$$\begin{array}{cc} RB_{0}^{-} & =\frac{\Delta SS_{0}^{-}}{SS_{0(ref)}}, \end{array}$$ (21)

$$\begin{array}{cc} RB_{1}^{-} & =\frac{\Delta SS_{1}^{-}}{SS_{1(ref)}}, \end{array}$$ (22)

$$\begin{array}{cc} RB_{1}^{+} & =\frac{\Delta SS_{1}^{+}}{SS_{1(ref)}}, \end{array}$$ (23)

where *SS*_0(*ref*)_ and *SS*_1(*ref*)_ are the prediction errors of the reference model for the non-events and events, respectively (*SS*_0(*ref*)_ + *SS*_1(*ref*)_ = *SS*_(*ref*)_).

The *RB* coefficients quantify the relative changes in the prediction error of the reference model. $RB_{0}^{+}$ and $RB_{1}^{+}$ are fractions by which the reference error was reduced, while $RB_{0}^{-}$ and $RB_{1}^{-}$ are fractions by which the reference error was increased in the corresponding outcome class.

The range of $RB_{0}^{+}$ and $RB_{1}^{+}$ is [0, 1], where 1 (100%) means a complete reduction of the reference error, and 0 means no error reduction. On the other hand, the range of $RB_{0}^{-}$ and $RB_{1}^{-}$ is [0, ∞). The higher their values, the greater the increase of the prediction error.

We also define the net *RB* coefficients stratified by outcome class as:

$$\begin{array}{cc} RB_{0} & =RB_{0}^{+}-RB_{0}^{-}, \end{array}$$ (24)

$$\begin{array}{cc} RB_{1} & =RB_{1}^{+}-RB_{1}^{-}. \end{array}$$ (25)

The range of *RB*_0_ and *RB*_1_ is (−∞, 1], where 1 means that the prediction error of the reference model was completely reduced, and 0 means that the prediction of the new model is only as good as that of the reference model. Negative values indicate that the prediction of the new model is worse than that of the reference model in the corresponding outcome class. The *RB*_0_ and *RB*_1_ coefficients express the net relative improvement of the prediction for non-events and events, respectively. Adding a perfect (and theoretical) new predictor to the reference model would reduce the overall prediction error. We would observe the following values of the *RB* coefficients: $RB_{0}^{+}=1$, $RB_{0}^{-}=0$, $RB_{1}^{-}=0$, and $RB_{1}^{+}=1$, and, further, *RB*_0_ = 1 and *RB*_1_ = 1.

### The U-smile plot and the U-smile method

**Fig 2:**
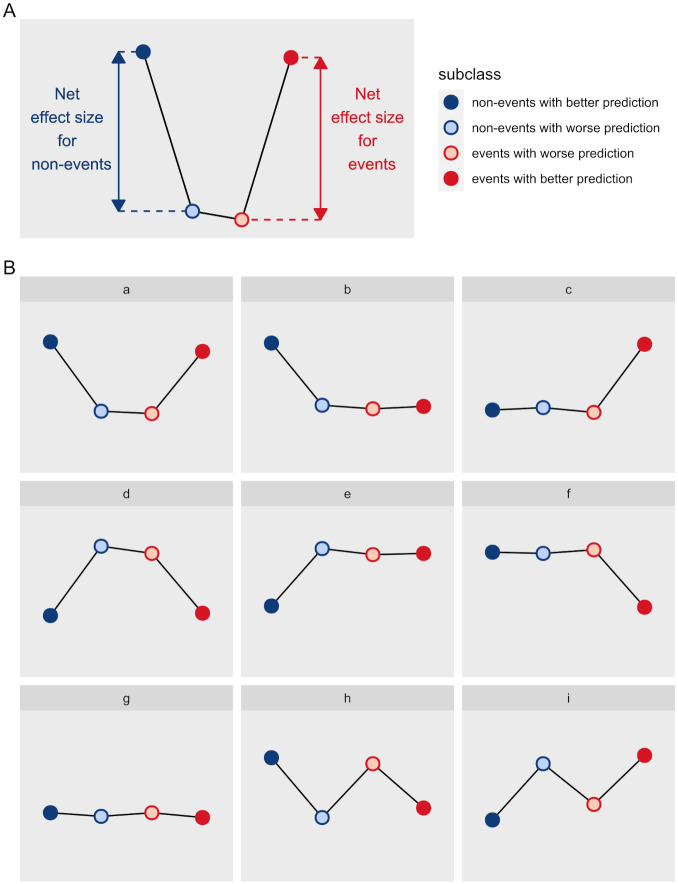
U-smile plot shapes. (A) The distance between the cilcles representing the non-event outcome class (blue cilcles) and the event class (red cilcles) on the U-smile plot are the net effect size of prediction improvement offered by a new marker. (B) Examples of possible shapes of the U-smile plot. Prediction improvement: (a) for both outcome classes, (b) only for the non-events, (c) only for the events. Prediction worsening: (d) for both outcome classes, (e) only for the non-events, (f) only for the events. Cilcles lying at an approximately constant level translate into no prediction improvement or worsening compared to the reference model (g). A zigzag indicates prediction improvement in one outcome class and prediction worsening in the other (h and i). As the shape of the plot is more important, any grid or scale is an unnecessary burden of information.

We propose the U-smile method (Fig 1 Step 4) to assess the improvement in the prediction of the reference model offered by the new predictor. This method quantifies prediction changes stratified by outcome subclass with improvement and worsening coefficients. These values are plotted in a specific order for the non-events and events, creating the U-smile plot. This plot effectively portrays the prediction change in the form of connected cilcles. U-smile plot does not always “smile”. The different shapes of the U-smile plot allow clear assessment and interpretation (Fig 2).

### Prediction improvement-worsening plot as a complement to the U-smile plot

**Fig 3:**
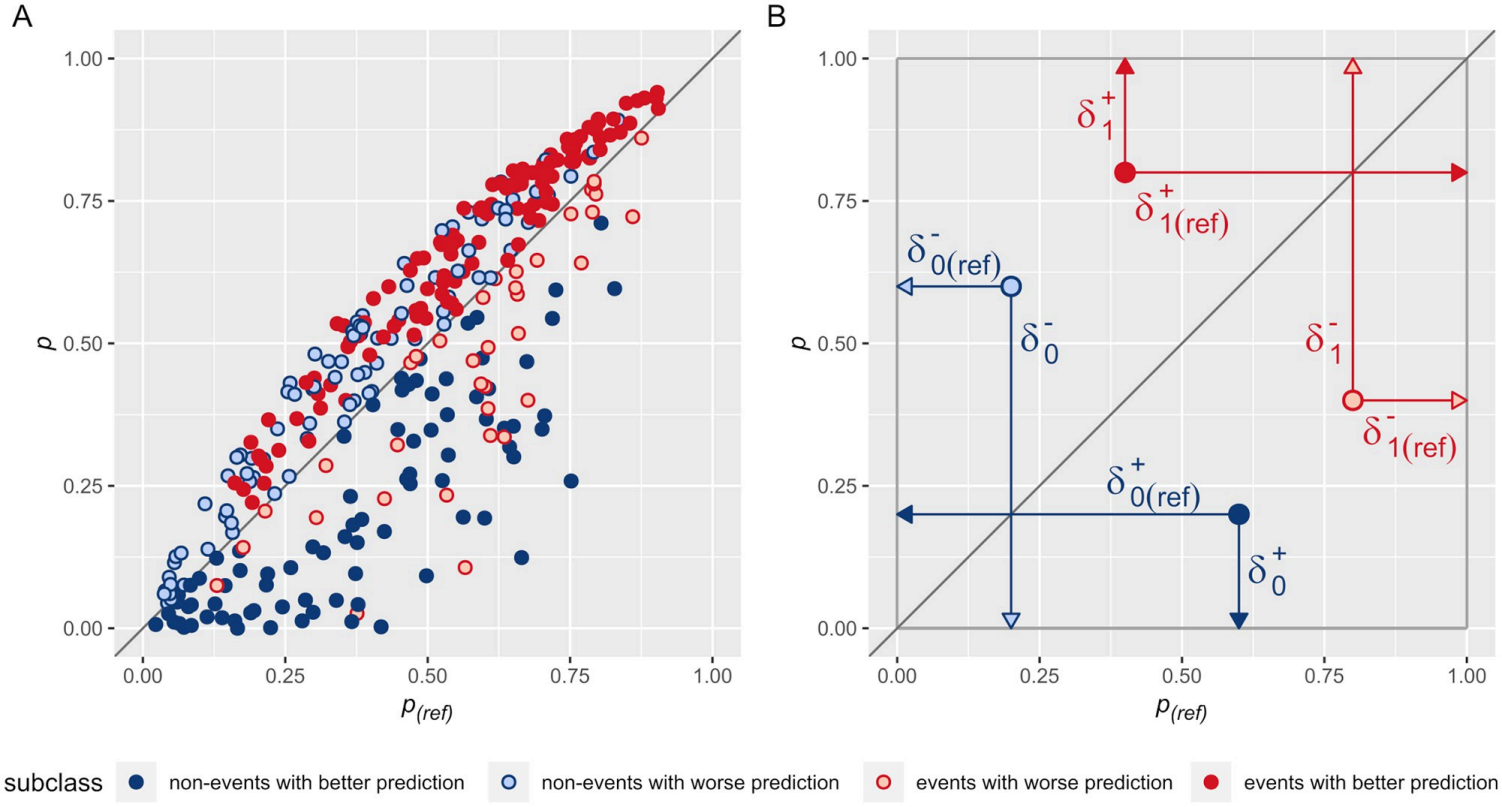
PIW plot with 4 subclasses stratification. Cross-tabulating changes in predicted probabilities with outcome class (i.e. non-event and event) across the identity line yields four subclasses of individuals. Subscripts _0_ and _1_ denote the non-events and the events, respectively. In contrast, according to the identity line, the superscripts (+) denote changes in a favourable direction (shorter residuals and better prediction of the new model) and the superscripts (-) denote changes in an unfavourable direction (longer residuals and worse prediction of the new model). Dark blue points represent the non-events with better prediction, light blue points—the non-events with worse prediction, dark red points—the events with better prediction, and light red points—the events with worse prediction. (A) A complete prediction improvement-worsening (*PIW*) plot. (B) Residuals of the reference model (*δ*_(*ref*)_) and the new model (*δ*) of an exemplary point for each outcome subclass.

The prediction-improvement Worsening (*PIW*) plot (Fig 3) visualises the position of the individuals relative to the probability of the reference model, *p*_(*ref*)_ (*X*-axis), and the probability of the new model, *p* (*Y*-axis) [31]. In essence, if a new model does not alter the probability prediction compared to the reference model, the corresponding individual falls on the identity line, *p*_(*ref*)_ = *p*. If the new model improves the predictive performance of the reference model, the darker red points for events and darker blue points for non-events on the *PIW* plot should diverge further from the identity line. On the other hand, light red and light blue points for individuals with worsened prediction should converge closer to the identity line.

Moving the points away from the identity line shows how close they are to the target probabilities, i.e. to the axis of the plot. The residuals of the model are the distances of the points from the target probabilities (0 for the non-events and 1 for the events) (Fig 3B). The vertical residuals correspond to the new model (along the *Y*-axis), and the horizontal residuals correspond to the reference model (along the *X*-axis). We take the squares of the residuals of the new and reference models to calculate the *BA* and *RB* coefficients. In determining the *BA* coefficient, we subtract the squares of the residuals, and in determining the *RB* coefficient, we additionally relate this difference to the squares of the residuals of the reference model. In this way, the *BA* and *RB* coefficients reflect the absolute and relative changes, respectively.

### Data

We used the Heart Disease dataset [32, 33], available from the UCI Machine Learning Repository [34] (accessed December 13, 2022). No sensitive or identifiable patient information such as names, addresses, contact details, etc. are available in this dataset. The dataset consists of four databases and contains 14 attributes. We combined all four databases into one raw dataset. Coronary artery disease, confirmed by coronary angiography, is the predicted event. It is defined as luminal narrowing >50% of any major coronary artery.

Only observations without missing values were included in the analysis (complete case analysis), and observations with resting blood pressure or serum cholesterol equal to zero were removed (incorrect values assumed). The obtained raw data set consisted of 661 observations: 303 observations (45.8%) from the Cleveland database, 261 (39.5%) from the Hungarian database, and 97 (14.7%) from the VA database. We analysed the following predictors:

- Age in years;
- Gender (1 = male, 0 = female);
- Type of chest pain (1 = typical angina, 2 = atypical angina, 3 = non-anginal pain, 4 = asymptomatic = reference category);
- Resting systolic blood pressure (in mmHg) on hospital admission;
- Cholesterol serum concentration in mg/dl;
- Fasting blood glucose concentration >120 mg/dl (1 = yes, 0 = no);
- Resting electrocardiographic (ECG) changes (0 = normal—reference category, 1 = ST-T wave abnormality: T wave inversions and/or ST elevation or depression >0.05 mV, 2 = probable or definite left ventricular hypertrophy according to Estes criteria);
- Maximum heart rate (in beats per minute) achieved during peak exercise on a treadmill;
- Exercise-induced angina (1 = yes, 0 = no);
- Exercise-induced ST depression relative to rest in mm.

The set of real variables included in the heart disease dataset is not sufficient to test the robustness of the method when expanding the models with variables of arbitrary distributions. In uninformative and informative scenarios, we generated independent random variables from some of the most common distributions in nature: the normal, uniform, exponential, Bernoulli, binomial and Poisson distributions. The six non-informative predictors were generated without stratification by outcome class, and are denoted by *Rnd* before the distribution name. The six informative predictors were generated with stratification by outcome class and are indicated by *Str Rnd* before the distribution name. The parameters of the generated random variables are shown in Table 2. The parameters set for generating data from the models in the non-informative scenario used fairly typical conditions. For example, a standardized normal distribution was chosen, and the Bernuli and binomial distributions reflect the class imbalances that often appear in the data. For the informative scenarios, the parameters were chosen so that the added variables were both very strong and distinkt, detectable as significant by the LRT test and DeLong’s test, e.g., for the normal distribution, remote means (10 and 12) were chosen, and quite weak, detectable only by the LRT test, e.g., for the Poisson distribution, the lambda was set to 1 and 1.6.

**Table 2:** Parameters of the independent random variables generated from theoretical probability distributions for validating the U-smile method. Data were generated to simulate a scenario when a non-informative predictor is added to the reference model (Random variables) and when an informative predictor is added to the reference model (Stratified random variables).

	Random variables	Stratified random variables	
Distribution	Overall	Non-events	Events
Normal	*N*(0, 1)	*N*(10, 2)	*N*(12, 2)
Uniform	*U*(0, 10)	*U*(0, 6)	*U*(2, 8)
Exponential	*exp*(λ = 1)	*exp*(λ = 0.5)	*exp*(λ = 1)
Bernoulli	*Bern*(*p* = 0.8)	*Bern*(*p* = 0.5)	*Bern*(*p* = 0.2)
Binomial	*Binom*(*n* = 6, *p* = 0.8)	*Binom*(*n* = 7, *p* = 0.6)	*Binom*(*n* = 7, *p* = 0.5)
Poisson	*Pois*(λ = 1)	*Pois*(λ = 1)	*Pois*(λ = 1.6)

We expect the non-informative predictors to produce approximately horizontal-shaped U-smile plots with values of the *BA* and *RB* coefficients close to zero, reflecting neither improvement nor worsening of prediction. On the contrary, we expect the informative predictors to produce smiling U-smile plots and positive values of the net coefficients. Furthermore, we generated age-dependent random variables from the normal distribution under the non-informative (*Rnd*) and informative (*Str Rnd*) scenarios to confirm that the negative effect of highly correlated predictors on model prediction will be visible in the U-smile plots. We restricted to a normal distribution due to the large number of new models in the dependent scenario. Unstratified variables were generated from the standard normal distribution. Stratified variables were generated from the distribution in a slightly weaker form than for the independent scenario, detectable as significant only by the LRT tests. We therefore assumed slightly closer means *N*(11, 2) for the events and from the distribution *N*(10, 2) for the events. These variables were generated from normal distributions with a predetermined Pearson’s correlation coefficient ranging from 0.1 to 0.9.

The raw dataset was randomly divided into training and test datasets: 331 observations in the training dataset (disease prevalence 47.4%) and 330 observations in the test dataset (disease prevalence 47.6%). The proportions of Cleveland, Hungarian and VA databases were reproduced from the raw dataset in the training and test datasets to ensure the best representation of the sample.

### Statistical analysis

The reference model was a logistic regression model with sex, age, systolic blood pressure (*SBP*) and total cholesterol (*Chol*) as the set of reference predictors, *X*. The outcome variable, *D*, *D* ∈ {0, 1}, was the presence of coronary disease in a patient. The reference model included the predictors of the Heart Disease dataset that are also included in the Framingham Risk Score [35] to simulate a practical approach. The reference model is hence given by:

$$\begin{array}{c} logitP\left(D=1|X\right)=\alpha_{0}+\alpha_{1}Sex+\alpha_{2}Age+\alpha_{3}SBP+\alpha_{4}Chol. \end{array}$$ (26)

In the independent scenario, we built 18 new models adding each independent candidate predictor *Y*_*j*_, *j* = 1, …, 18, to the reference model: six real predictors from the Heart Disease dataset, six random variables without stratification by outcome class, and six random variables stratified by outcome class. In addition, in the dependent scenario, we built 18 new models by adding random variables correlated with age (found in the reference model): nine without stratification and nine stratified by outcome class. Thus, each new model is given by:

$$\begin{array}{c} logitP\left(D=1|X,Y_{j}\right)=\beta_{0}+\beta_{1}Sex+\beta_{2}Age+\beta_{3}SBP+\beta_{4}Chol+\beta_{5}Y_{j}. \end{array}$$ (27)

All models were fitted on the training dataset and applied to the test dataset for validation.

By definition, *I* [25] consists of the *I* coefficients stratified by outcome subclass:

$$\begin{array}{cc} I_{0}^{+} & =\frac{n_{0}^{+}}{n_{0}}, \end{array}$$ (28)

$$\begin{array}{cc} I_{0}^{-} & =\frac{n_{0}^{-}}{n_{0}}, \end{array}$$ (29)

$$\begin{array}{cc} I_{1}^{-} & =\frac{n_{1}^{-}}{n_{1}}, \end{array}$$ (30)

$$\begin{array}{cc} I_{1}^{+} & =\frac{n_{1}^{+}}{n_{1}}, \end{array}$$ (31)

where $n_{0}^{+}$ and $n_{0}^{-}$ are the numbers of non-events with better and worse prediction, respectively ($n_{0}^{+}+n_{0}^{-}=n_{0}$), and $n_{1}^{-}$ and $n_{1}^{+}$ are the numbers of events with worse and better prediction, respectively ($n_{1}^{-}+n_{1}^{+}=n_{1}$). The coefficients *I* range from 0 to 1 and indicate a proportion of individuals with a prediction change in each subclass (i.e. a proportion of reclassified individuals according to Pencina’s definition of reclassification). Then

$$\begin{array}{c} I=I_{0}^{+}-I_{0}^{-}+I_{1}^{+}-I_{1}^{-}=I_{0}+I_{1}, \end{array}$$ (32)

where *I*_0_ and *I*_1_ are the net coefficients for the non-events and events, respectively. The range of *I*_0_ and *I*_1_ is [−1, 1], and the range of the *I* is [−2, 2]. Higher values indicate more correctly reclassified individuals, and thus an improvement in prediction relative to the reference model.

We calculated the *BA*, *RB* and *I* coefficients for all new models and plotted them on the U-smile plots. The graphical assessment also included the new models’ *ROC* curves and the *PIW* plots. We compared each new model with the reference model using the *LRT*. The Δ*AUC* between the new and reference models was assessed using the DeLong’s test for two correlated *ROC* curves [36]. A significance level 0.05 was assumed for the *LRT* and the DeLong’s test for two correlated *ROC* curves. The prediction improvement evaluation using the U-smile method was repeated for the models derived from the test dataset.

All analyses were performed using statistical software R (v. 4.2.2) [37] and RStudio (v. 2023.6.0.421) [38].

## Results

**Fig 4:**
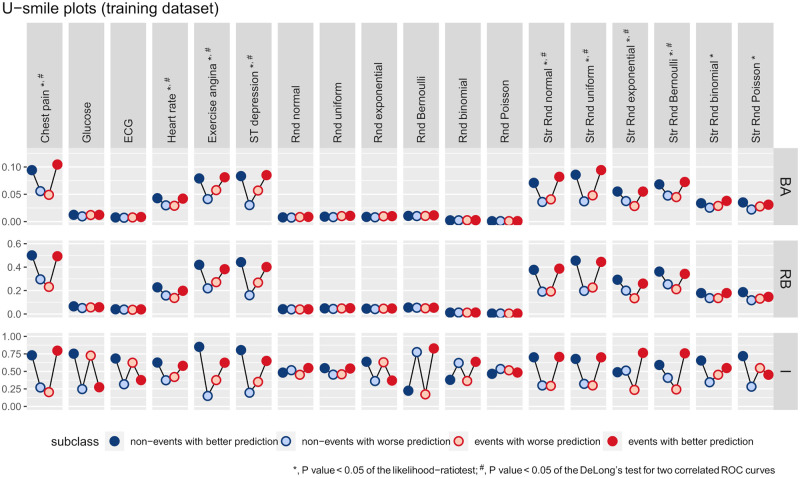
The U-smile plots of the *BA*, *RB* and *I* coefficients for each new model derived from the training dataset under the independent scenario.

Fig 4 shows the U-smile plots of the *BA*, *RB* and *I* coefficients for the models derived from the training dataset. The U-smile plots of the *BA* and *RB* coefficients take the shape of a smile or an approximately horizontal line. In contrast, the U-smile plots of the *I* coefficients have various shapes. Table 3 shows the values of the net *BA*, *RB* and *I* coefficients, and Table 4 displays the results of the *LRT* and the *ROC* curve analysis.

**Table 3:** Values of the net *BA*, *RB* and *I* coefficients stratified by outcome class for 18 new models derived from the training and test datasets under the independent scenario. The reference model was expanded by six real predictors from the Heart Disease dataset, 6 non-informative random variables (without stratification by outcome class), and 6 informative random variables (stratified by outcome class).

	Training dataset						Test dataset					
New model	*BA*_0_	*BA*_1_	*RB*_0_	*RB*_1_	*I*_0_	*I*_1_	*BA*_0_	*BA*_1_	*RB*_0_	*RB*_1_	*I*_0_	*I*_1_
**Real predictors**												
Chest pain	0.039	0.056	0.205	0.262	0.460	0.592	0.049	0.062	0.269	0.258	0.526	0.541
Glucose	0.003	0.000	0.014	0.001	0.506	-0.452	-0.003	0.000	-0.016	-0.002	0.468	-0.605
ECG	0.001	0.001	0.003	0.003	0.310	-0.108	0.002	0.000	0.012	0.000	0.410	-0.121
Heart rate	0.013	0.013	0.070	0.061	0.253	0.159	0.013	0.032	0.070	0.130	0.202	0.236
Exercise angina	0.038	0.024	0.201	0.111	0.701	0.248	0.041	0.053	0.228	0.220	0.688	0.287
ST depression	0.053	0.028	0.282	0.133	0.609	0.299	0.025	0.050	0.140	0.207	0.434	0.363
**Random variables**												
Rnd normal	0.000	0.000	0.002	0.001	-0.034	0.096	0.001	0.001	0.006	0.003	0.006	0.083
Rnd uniform	0.001	0.000	0.004	0.002	0.092	0.083	0.003	-0.006	0.014	-0.023	0.179	-0.172
Rnd exponential	0.001	0.000	0.004	0.001	0.276	-0.261	-0.002	0.001	-0.012	0.005	0.179	-0.210
Rnd Bernoulli	0.000	0.001	0.002	0.005	-0.552	0.656	-0.004	0.001	-0.019	0.004	-0.734	0.694
Rnd binomial	0.000	0.000	0.000	0.001	-0.241	0.274	0.000	0.000	0.000	0.001	-0.237	0.325
Rnd Poisson	0.000	0.000	-0.001	0.000	-0.069	-0.032	0.000	0.000	0.000	0.000	-0.168	-0.019
**Stratified random variables**												
Str Rnd normal	0.035	0.041	0.186	0.195	0.402	0.414	0.012	0.054	0.067	0.225	0.364	0.439
Str Rnd uniform	0.049	0.046	0.259	0.219	0.356	0.401	0.029	0.045	0.160	0.188	0.434	0.338
Str Rnd exponential	0.017	0.027	0.093	0.125	-0.023	0.529	0.004	0.031	0.020	0.128	-0.098	0.529
Str Rnd Bernoulli	0.020	0.028	0.109	0.131	0.184	0.516	0.023	0.016	0.128	0.067	0.179	0.376
Str Rnd binomial	0.008	0.009	0.043	0.043	0.310	0.096	0.026	-0.002	0.144	-0.008	0.595	-0.057
Str Rnd Poisson	0.013	0.003	0.069	0.015	0.437	-0.096	0.015	0.004	0.085	0.018	0.526	-0.146

ECG, resting electrocardiographic changes

**Table 4:** Comparisons of the reference model with each new model derived from the training dataset under the independent scenario. The reference model was expanded with six real predictors from the Heart Disease dataset, six non-informative random variables (without stratification by outcome class), and six informative random variables (stratified by outcome class). Shown are the values of the *AUC* of all new models. Δ*AUC* shows the difference in *AUC* relative to the reference model. The *AUC* of the reference model is 0.758. The DeLong’s test for two correlated *ROC* curves was used to asses Δ*AUC*.

New model	AUC	ΔAUC	ΔAUC *P* value	LRT *P* value
**Real predictors**				
Chest pain	0.853	0.095	0.000	0.000
Glucose	0.761	0.002	0.689	0.103
ECG	0.760	0.002	0.633	0.696
Heart rate	0.789	0.031	0.023	0.000
Exercise angina	0.825	0.067	0.000	0.000
ST depression	0.843	0.085	0.000	0.000
**Random variables**				
Rnd normal	0.757	-0.001	0.765	0.355
Rnd uniform	0.758	0.000	0.986	0.306
Rnd exponential	0.758	0.000	0.931	0.248
Rnd Bernoulli	0.759	0.001	0.839	0.202
Rnd binomial	0.757	-0.001	0.318	0.782
Rnd Poisson	0.758	0.000	0.892	0.917
**Stratified random variables**				
Str Rnd normal	0.843	0.085	0.000	0.000
Str Rnd uniform	0.862	0.104	0.000	0.000
Str Rnd exponential	0.810	0.052	0.002	0.000
Str Rnd Bernoulli	0.814	0.056	0.002	0.000
Str Rnd binomial	0.778	0.020	0.108	0.000
Str Rnd Poisson	0.776	0.018	0.123	0.000

ROC, receiver operating characteristic; AUC, area under the ROC curve; LRT, likelihood-ratio test; ECG, resting electrocardiographic changes.

The results of the U-smile method for the generated random predictors are consistent with our assumptions. The U-smile plots of the *BA* and *RB* coefficients for the new models with the non-informative predictors (Rnd + distribution) are horizontal lines close to zero. This means that the predictions of the reference and new models are only slightly different. The *I* coefficients express the number of prediction changes, regardless of how small or large they are. Therefore, we observe a high variability of the *I* coefficients. In particular, the U-smile plot of the *I* coefficients indicates prediction improvement for the new model extended with Rnd uniform. However, for Rnd Poisson it indicates prediction worsening. The results of the *LRT* and the DeLong’s test for two correlated *ROC* curves are above the assumed significance level. These are consistent with the results of the U-smile method.

The U-smile plots of the *BA* and *RB* coefficients for the new models with the informative predictors (Str Rnd + distribution) have the shape of a smile or the letter U, thus indicating prediction improvement for both outcome classes. However, in the case of Str Rnd binomial and Str Rnd Poisson, the smile is less pronounced than in the case of the other stratified predictors. These random predictors were generated in such a way that adding them to the reference model only slightly increases the *AUC* of the reference *ROC* curve. The *LRT* results are below the assumed significance level for the six stratified predictors, consistent with the shape of the U-smile plots.

The results of the DeLong’s test for two correlated *ROC* curves are below the assumed significance level for all stratified predictors except for Str Rnd binomial and Str Rnd Poisson. This agrees with the less apparent smile of the U-smile plots of these predictors. The U-smile plots of the *I* coefficients show prediction worsening for Str Rnd Poisson for the events and slight prediction worsening for Str Rnd exponential for the non-events. However, they show prediction improvement for the other stratified predictors.

When the reference model was expanded by real predictors of the Heart Disease dataset, all methods produced concordant results. The U-smile plots of the *BA*, *RB* and *I* coefficients smile for chest pain, heart rate, exercise angina, and ST depression. However, the smile of the U-smile plots for heart rate is less apparent. The *LRT* and the DeLong’s test for two correlated *ROC* curves yielded results below the significance level for chest pain, heart rate, exercise angina, and ST depression. Meanwhile, for glucose and ECG, the U-smile plots of the *BA* and *RB* coefficients have the shape of a horizontal line, while the U-smile plots of the I coefficients have the shape of a zigzag.

**Fig 5:**
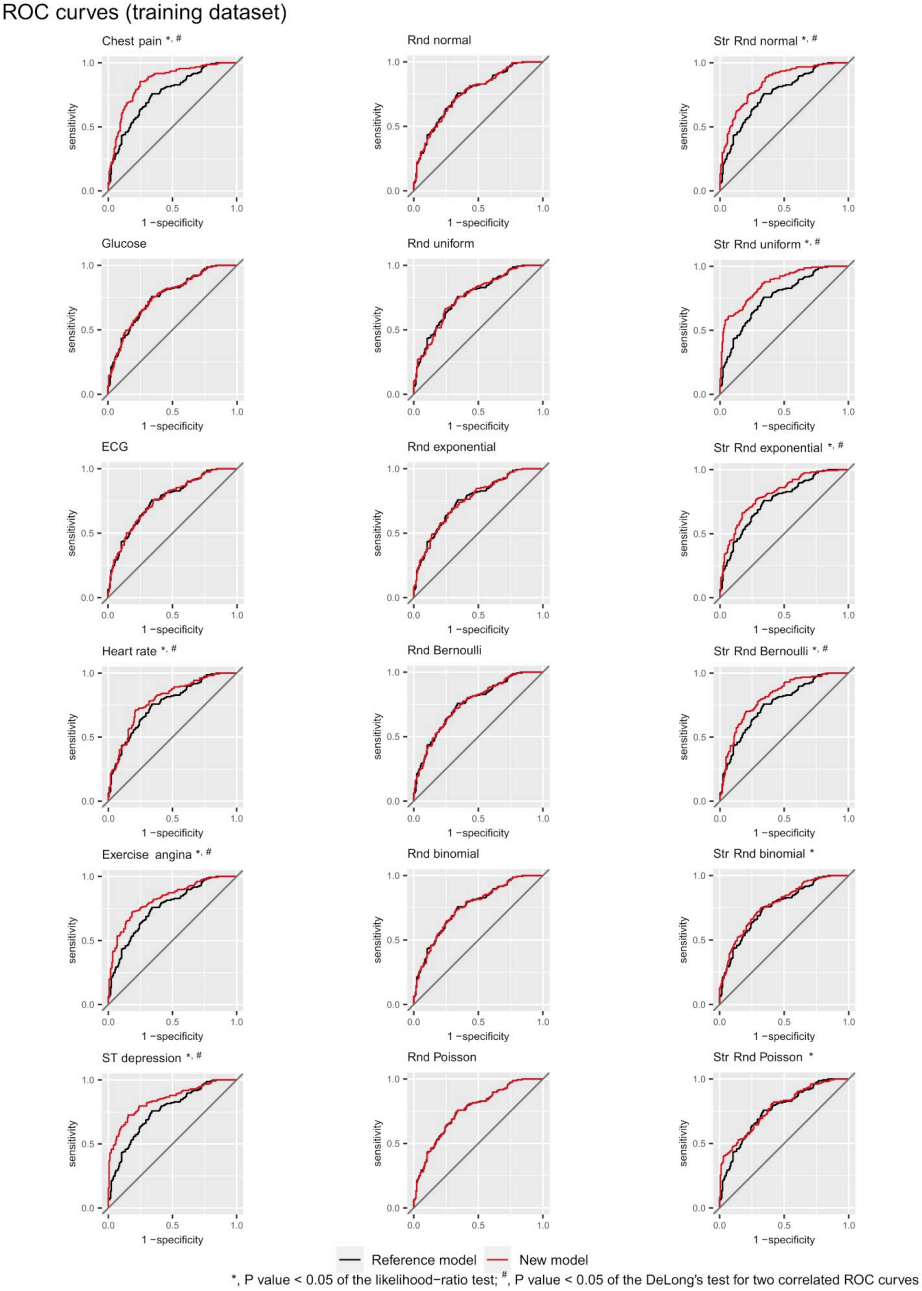
The *ROC* curves of the reference and new models derived from the training dataset under the independent scenario.

The *AUC* of the reference model is 0.758, and the values of the *AUC* of the new models are provided in Table 4. Fig 5 and S2 Fig show the *ROC* curves of the reference and new models derived from the training and test datasets, respectively.

**Fig 6:**
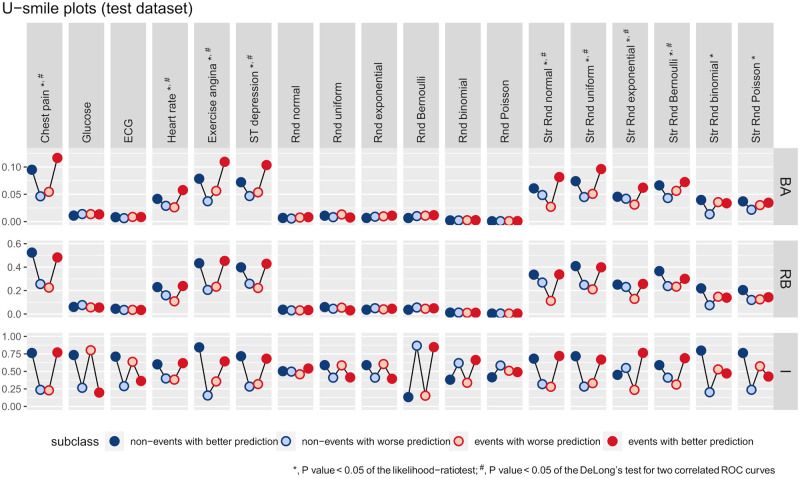
The U-smile plots of the *BA*, *RB* and *I* coefficients for each new model derived from the test dataset under the independent scenario.

**Fig 7:**
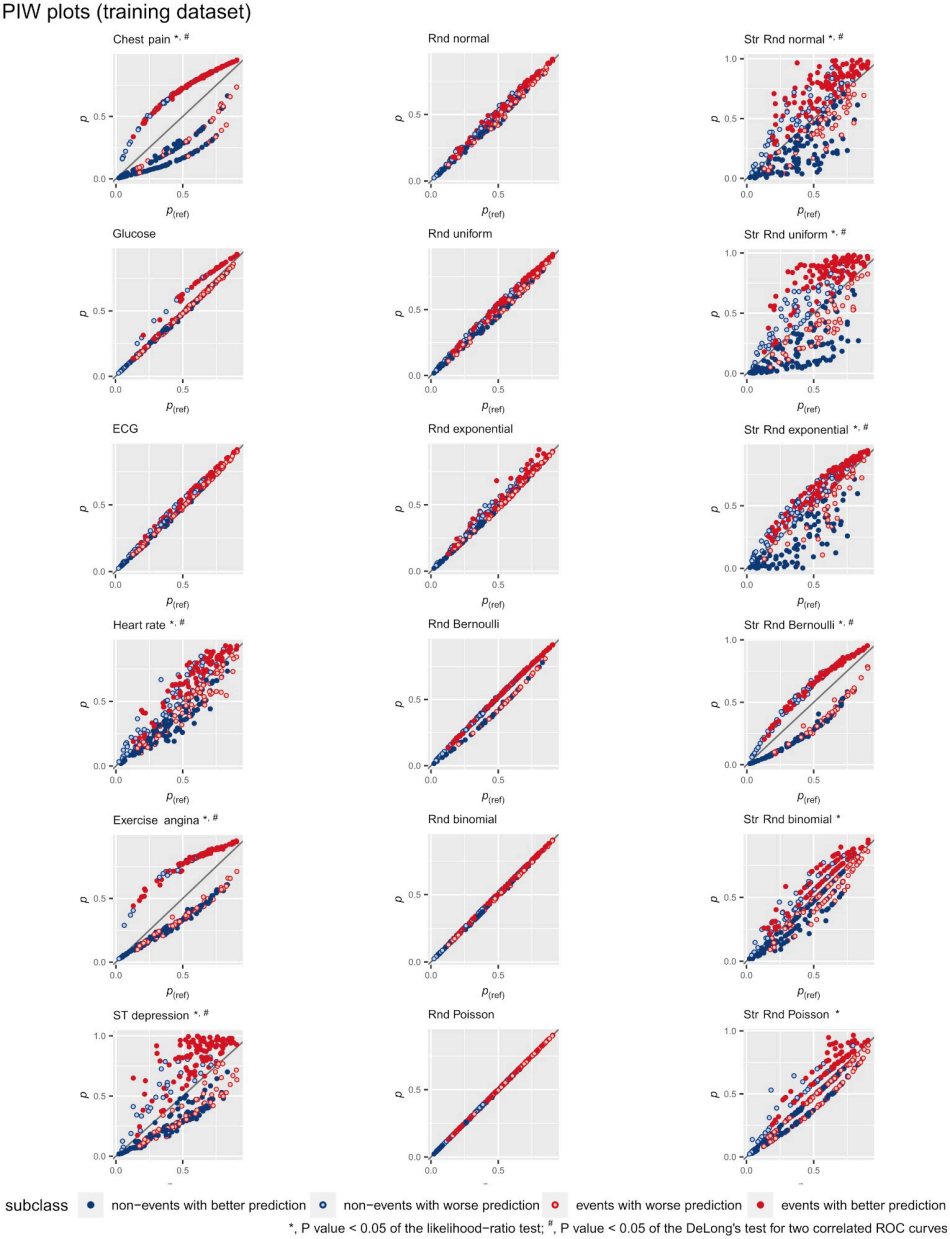
The prediction improvement-worsening (*PIW*) plots for each new model derived from the training dataset under the independent scenario.

Fig 6 shows the U-smile plots of the *BA*, *RB* and *I* coefficients for the models derived from the test dataset. The values of the *BA*, *RB* and *I* coefficients stratified by outcome subclass for models derived from the training and test datasets are provided in Tables 1 and 2 of S1 Appendix, respectively. Fig 7 and S1 Fig show the *PIW* plots of the reference and new models derived from the training and test dataset, respectively. S3 Fig shows the U-smile plots of the *BA*, *RB* and *I* coefffcients for models derived from the training and test datasets, with additional random variables correlated with age. The higher the Pearson correlation coefffcient, up to 0.8, the less the U-smile plot smiles until the model loses its resistance to over-correlation and becomes less stable. This effect is present in the models derived from the training dataset and is even better visible in the models derived from the test dataset. In the latter case, the smile disappears and the predictions of the new model are weaker than those of the reference model.

## Discussion

We present a new and validated approach for graphical and quantitative assessment of the prediction improvement gained by adding a new marker to a set of reference markers for predicting a binary outcome: the U-smile method. By design, the U-smile plot smiles when the new marker improves the prediction of the reference model for both outcome classes: the larger the smile, the greater the prediction improvement. No smile (a horizontal line) means no improvement, while an asymmetric smile means improvement for one outcome class. A zigzag smile indicates improvement for one outcome class but worsening for the other.

To confirm the accuracy of the U-smile method in generating truly smiling or non-smiling plots, we performed a series of tests using real and generated variables under both informative and non-informative scenarios. The U-smile method correctly identiffed both non-informative and informative predictors, regardless of the tested data distribution. Visual evaluation of the U-smile plots of the *BA* and *RB* coefffcients allowed immediate interpretation of the results. The U-smile plots of the non-informative predictors took the shape of horizontal lines located at the bottom of the plots and did not obscure the smiling U-smile plots of the informative predictors.

We also used random predictors with varying degrees of multicollinearity. When the random data were correlated with informative parameters, the generated U-smile plots smiled like for the informative data. This suggests that U-smile plots are able to detect predictive information hidden in the analysed data.

### Comparison with ROC curves

Due to frequent overlap, a clear graphical comparison of multiple candidate predictors is not always possible with *ROC* curves (Fig 5). However, such a comparison is easy when many candidate predictors are presented side by side on the U-smile plots. Each predictor has an individual U-smile plot with no overlap.

The predictors offering the greatest prediction improvement can be clearly and quickly identiffed. Therefore, the U-smile method provides deeper insight into prediction improvement than the *ROC* curves. In our study, for example, the net effect size of prediction improvement by ST-segment depression is approximately twice as large for the non-events than for the events. Similar information is not available from the *ROC* curves.

The *ROC* curve measures the model’s ability to discriminate between positive and negative outcomes. The *AUC* estimates how well the model predictions are ordered and is not sensitive to their values. It is the main limitation of this method. Δ*AUC* for different predictors or models may not fully capture the improvement in the actual predicted probabilities and may not provide a complete picture of the model performance.

Not considering the absolute values of the predictions by the *ROC* curve has its consequence, two models with different predicted probabilities but the same rank order would have identical *AUC*s. On the contrary, the U-smile method evaluates the performance of different models and predictors based on the magnitude of the probability changes relative to the reference model. We obtained a pair of new models with the same Δ*AUC* (a pair of ST depression and Str Rnd normal, as shown in Table 4). However, their *BA* and *RB* coefficients (Table 1 in S1 Appendix), and U-smile plots (Fig 4) were distinct. Various models with identical *AUC*s may have different shapes of their *ROC* curves. The U-smile method describes each new model by four sets of three different coefficients (*BA*, *RB*, and *I*) and one summary U-smile plot. The probability of two different models having identical all twelve coefffcients and U-smile plots is very low, if possible.

### The PIW and U-smile plots

The *PIW* plot visualises the position of the individuals relative to the identity line, representing no change in probability between the reference model and the new model [31] and shows both the magnitude and number of changes for each subclass (Fig 7 and S1 Fig). On the *PIW* plot, we can observe the nature of the variable added to the model. For example, chest pain is a 4-category variable (3 subject categories and 1 reference category), therefore the points of the graph are arranged along these categories. Exercise angina is a 2-category variable, so the points of the *PIW* plot form 2 curves, and ST depression is continuous, giving a cloud of points not forming specific lines.

The U-Smile and *PIW* plots are complementary. The points representing individuals in each subclass in the *PIW* plot are aggregated and represented by cilcles of the same colour in the U-smile plot. Subclass-specific points in different colours on the *PIW* plot represent the number of individuals, summarised quantitatively in the *I* coefficients. The upward and downward distances of the points from their target probabilities are summarised by the *BA* and *RB* coefficients.

The *PIW* plot allows more precise identification of an individual subject and whether the new parameter improves, worsens or does not change the prognosis. The U-smile plot, on the other hand, gives a general impression based on averaged values specific to subclasses.

### U smile plots for the I coefficient

We compared the results of the U-smile method with those of the *I*. Replacing the *BA* or *RB* coefficients with the *I* components in the U-smile plot may result in different shapes. The *I* may falsely indicate positive results by producing a total smile, i.e. for non-events and events, or a partial smile for only one class. Some examples are Rnd uniform for both outcome classes, Rnd Bernoulli for the events, or false negatives for Rnd Poisson for both outcome classes.

The *BA* and *RB* coefficients quantify the magnitude of prediction changes, while the *I* expresses the number of these changes. This explains the difference between these two methods. Adding a new marker to the reference model almost always changes its predictions, however small these changes may be for the non-informative predictors. Therefore, we almost always have: $I_{0}^{-}+I_{0}^{+}+I_{1}^{+}+I_{1}^{-}=\frac{n_{0}^{-}+n_{0}^{+}}{n_{0}}+\frac{n_{1}^{+}+n_{1}^{-}}{n_{1}}=1+1=2$. These erroneous values of the *I* coefficients, and their U-smile plots suggest that the *I* alone should not be relied upon as a measure of prediction improvement. The *I* should be interpreted in the *BA* and *RB* coefficients context. We suggest treating *I* coefficients as complementary rather than competing with the *BA* and *RB* coefficients. In this way, we can better understand the factors that contribute to the prediction of the model.

### Collinearity

Collinearity affects the performance and interpretation of predictive models by inflating the variance of the coefficients, making them unstable and unreliable. Adding similar or redundant information can increase the degree of collinearity and exacerbate the problem. If the correlation between theoretically independent predictors is high enough, reliable model fitting and interpretation of results may be impossible. Such models behave erratically in response to small changes in the data or in the procedure used to build the predictive models [39, 40].

We used random predictors generated from normal distributions and correlated with age already included in the reference model. The U-smile plots became flatter as the correlation increased, indicating that adding a fairly strongly correlated variable to the reference model becomes less favourable. For Pearson correlation coefficients above 0.8 and variables stratified by outcome class, the logistic regression models became less stable, and consequently, the U-smile plot lost its resistance to over-correlation. The U-smile method, when employed for variable selection, may help mitigate the risk of collinearity in predictive models to a certain degree. If collinearity is severe, it may not indicate that one of the highly correlated parameters should be excluded from the model. Currently, the U-smile plot visualises the *BA*, *RB* and *I* coefficients. In the future, however, the U-smile plot will integrate coefficients obtained from alternative techniques to represent different subclasses of individuals. Some of these methods may be effective in removing highly correlated variables.

This study used the simplest form of logistic regression without regularisation to assess collinearity. A U-smile plot can also be constructed for model-building methods that use regularisation techniques that are more robust to collinearity, such as least absolute shrinkage and selection operator (LASSO), ridge regression [41] or other suitable alternatives. Another potentially important application of the U-smile method is the graphical and transparent comparison of the range of robustness to collinearity of different prediction methods using U-smile plots.

### Reproducibility

The reproducibility of a computational method goes beyond simply producing identical results for the same data. It also includes the ability to reproduce results using the same code and data generated in similar scenarios, but with different data points.

We have used rigorously tested and validated mathematical algorithms and codes for the U-smile method. Through numerous simulations, parameter changes and repeated analyses we have consistently obtained identical results, demonstrating the computational reproducibility of our algorithms. The data are not shown, but are available through code-based generation at https://github.com/kbkubiak/U-smile folder: code. File 01 allows users to set parameters for individual distributions and generate data accordingly, while File 02 enables users to perform analyses and view the results plotted.

By generating independent random variables with different distributions, we have demonstrated the consistency of the U-smile method. For non-informative predictors, the U-smile plots were approximately horizontal, with the *BA* and *RB* coefficients close to zero. In contrast, for informative predictors, the U-smile plots smiled, and the net *BA* and *RB* coefficients were positive. When random variables added to the reference model were correlated with a variable in the reference model, the U-smiles smiled again for weak correlations and then consistently disappeared for moderate correlations.

We observed these effects in all repeated simulations and under various analysed scenarios, i.e. non-informative versus informative and independent versus dependent. Overall, the U-smile method ensures high reproducibility of prediction for the same or similar types of parameters.

### Connection with a proper measure: The Brier score

There are few methods for assessing model performance that are both quantitative and stratified. In an imbalanced scenario, the stratified Brier score (*BS*) was proposed for the non-events and the events [23, 24, 26]. Like the *LRT* and *ROC* curves, the *BS* is sensitive to imbalance [42, 43]. While the *BS* describes the average prediction accuracy, Δ*BS* measures the average change in the prediction, and the Brier skill score (*BSS*) quantifies the relative change in the prediction compared to the reference prediction. The connection between the net *BA* and net *RB* coefficients and the *BS* is obvious. Clearly, if we define $w_{n0}=\frac{n_{0}}{n}$ and $w_{n1}=\frac{n_{1}}{n}$ as weights, then:

$$\begin{array}{c} w_{n0}\cdot BA_{0}+w_{n1}\cdot BA_{1}=BS_{\left(ref\right)}-BS=\Delta BS, \end{array}$$ (33)

where Δ*BS* is the difference between the Brier score of the reference model, *BS*_(*ref*)_, and the Brier score of the new model, *BS* (for derivation see Eq (1) in S1 Appendix).

Moreover, if we define $w_{s0}=\frac{SS_{0(ref)}}{SS_{\left(ref\right)}}$ and $w_{s1}=\frac{SS_{1(ref)}}{SS_{\left(ref\right)}}$ as weights, then:

$$\begin{array}{c} w_{s0}\cdot RB_{0}+w_{s1}\cdot RB_{1}=1-\frac{BS}{BS_{\left(ref\right)}}=BSS, \end{array}$$ (34)

where *BSS* is the Brier skill score (for derivation see Eq (2) in S1 Appendix).

Regarding propriety, the *BA* coefficients add up to the Δ*BS* that is proper [30], and the *RB* coefficients add up to the *BSS* that is asymptotically proper [44]. Propriety is a desired feature of a performance metric since adding a superfluous predictor to the reference model does not increase the values of the proper measures but can increase the *I* [30]. On the other hand, the size of the area under the *ROC* curve is referred to as a semi-proper measure [45]. However, a detailed discussion of propriety is a separate and extensive topic [46, 47] beyond the scope of this paper.

### When does the U-smile plot smile and when does it not?

There are no specific values of the *BA* and *RB* coefficients that clearly separate flat U-smile plots from truly smiling U-smile plots. For this purpose we use the *LRT*, which is simple, informative and commonly used. Like the *BA* and *RB* coefficients, the *LRT* is based on the magnitude of the residuals of the models being compared. As with other tests, increasing the sample size of the study usually produces significant differences. So it is not the shape of the smile itself, but the sample size that might affect the outcome of the *LRT* and the assessment of whether a less smiling plot is significantly smiling or not.

### Why are two separate coefficients that produce similar U-smile plots introduced?

The *BA* and *RB* coefficients have the same numerator (Δ*SS*) but different denominators and interpretations. By dividing Δ*SS* by the number of individuals in a given class, the *BA* reflects the average absolute change in prediction due to a new parameter in class 0 (non-event) or 1 (event). In contrast, for *RB*, Δ*SS* divided by *SS*_*ref*_ for the corresponding class indicates how the new parameter affects the error relative to the residual *SS*.

Our preliminary analysis reveals that *BA* and *RB* coefficients exhibit distinct behaviours with unbalanced data, resulting in non-parallel U-smile curves. Balanced or nearly balanced data have similar denominator values, i.e. *n*_0_ and *n*_1_ for *BA*, and *SS*_0(*ref*)_ and *SS*_1(*ref*)_ for *RB*. In epidemiological and clinical studies, balanced data with comparable or equal numbers of people in both the non-event and event classes are rare and often considered exceptional, for example, in studies with exact matching. In contrast, prospective studies with consecutive enrolment of patients are more likely to be unbalanced. Our observations show that data imbalance leads to increasing differences in the shape of the U-smile plots for the *BA* and *RB* coefficients. In such cases, these coefficients are complementary rather than alternative indicators. This highlights the importance of considering both *BA* and *RB* for a more comprehensive understanding of various properties in newer binary classification and prediction methods using the U-smile approach.

### Study limitations and remaining questions

As already mentioned, we investigated a fairly balanced scenario with disease prevalence close to 50% and found that the U-smile plots of the *BA* and *RB* coefficients behave similarly. However, the numbers of non-events (*n*_0_) and events (*n*_1_) are unequal in many studies. In such cases, many prediction models (including logistic regression models) may suffer from an imbalance between the prediction errors of individual classes *SS*_0(*ref*)_ and *SS*_1(*ref*)_ [48]. The problem of prediction error imbalance is often assessed using the *BS*, which is related to the *BA* and *RB* coefficients [49]. Therefore, the U-smile plots of the *BA* and *RB* coefficients can be particularly useful in models built on imbalanced data. In such cases with unequal class sizes and prediction errors, the resulting U-smile plots of the *BA* and *RB* coefficients are likely to divert. This is a broad but important topic. However, due to space limitations, it is not covered in this paper. This issue is the subject of another ongoing investigation.

Various statistical methods and parameters may assess the prediction improvement for a binary outcome. Some of the commonly used methods are the net reclassification improvement (*I*), integrated discrimination improvement [25], decision curve analysis [50], calibration slope and intercept [16, 51], *LASSO* [19], *AUC* of the *ROC* curve, or the above described *BSS* and Δ*BS*.

The *I* coefficients measure the proportion of individuals correctly reclassified into higher or lower risk categories by the new model compared with the reference model. The integrated discrimination improvement examines the difference in average predicted probabilities between the new and reference models, stratified by the observed outcome. The decision curve analysis plots the net benefit of using the new model versus the reference model over a range of threshold probabilities for making a decision based on the predicted outcome. The *AUC* measures the ability of the model to discriminate between individuals who experience the outcome and those who do not, regardless of the threshold probability chosen. The calibration slope and intercept quantify the agreement between the predicted probabilities and the observed outcomes by comparing the average predicted probability and the observed outcome in groups of individuals. *LASSO* is a regression analysis method that performs both variable selection and regularisation by shrinking the regression coefficients and reducing some of them to zero. This helps to improve the prediction accuracy and interpretability of the resulting statistical mode. A set of diagnostic performance based on confusion matrix like sensitivity, specificity, accuracy, positive and negative predictive values or F1 score are another example [52].

All of these methods and parameters can be used to evaluate the predictive improvement of a new marker in different ways, depending on the research question, type of data and clinical context. The U-smile method has some similarities with several of the above methods. It incorporates the *I* coefficient and combines it with two other coefficients proposed by us, the *BA* and *RB*, and the novel graphical presentation in the form of a smile plot. However, due to space limitations, we have limited the direct comparison of the U-smile method with the *ROC* curve and its *AUC*, used the *I* coefficients, and found a relation between *BA* and Δ*BS*, and between *RB* and *BSS*. A comparison with other techniques that could be used to analyse the improvement in prediction obtained by adding a new marker to a reference method is necessary, interesting and important, and it deserves further investigation.

Utility measures how diagnostic tests improve health outcomes by informing clinical decisions, such as starting or stopping treatment for certain patients, while taking into account the consequences of wrong decisions. Based on this description, we have not directly tested the utility of the U-smile method and its impact on clinical outcomes. A separate study that compares the results of the U-smile method in improving prediction with real-world outcomes should be considered to explore this issue.

### Potential applications of the U-smile method

The U-smile plots help to compare candidate markers added to the reference model and evaluate their usefulness for prediction improvement. These plots can also visualise, explain and help understand the process of stepwise selection of variables into a prediction model. Once interesting models have been identified, the *PIW* plots can be used to deepen the analysis and explore where each individual falls relative to the prediction made by the reference and new models.

The *BA*, *RB* and *I* coefficients, together with the U-smile plot, allow for a more detailed evaluation of different prediction models. Our current investigation focuses on applying the U-smile method to nested models. Theoretical considerations suggest the method might be applicable to non-nested settings as well. However, the applicability of the U-smile approach to non-nested settings remains to be explored.

We have presented the *BA* and *RB* coefficients and the U-smile plot by comparing two logistic regression models. However, any other type of analysis that predicts binary values, such as neural networks or decision trees, can be used for the same purpose. The result of such an analysis yields a value in the range (0, 1) or, if in another range, might be transformable to that range, e.g. by min-max normalisation or S-function normalisation.

The proposed approach of dividing the studied individuals into four subclasses and displaying them on the U-smile plot can easily be applied to other parameters besides the *BA*, *RB* and *I* coefficients. For example, the U-smile plot can show the results of agreement between the two methods, as described by Cohen’s Kappa [53]. It can accompany descriptors such as diagnostic odds ratios, sensitivity and specificity values, accuracy, F1-score, and other similar measures based on the confusion matrix. Overall, the U-smile plot is a versatile tool that can be used to visualise and compare different measures and coefficients in a clear and concise manner.

Predictive forecasting and modelling are not unique to medicine and the health sciences. Optimisation of these processes based on similar approaches is used in economics, physical sciences, chemical sciences, meteorology, design and operation of engineering systems or processes, and many other fields. The U-smile method can compare the prediction performance in all of them. This method provides researchers and practitioners with a novel and multipurpose tool to better understand how new markers are selected and how they affect prediction accuracy.

## Conclusions

The proposed U-smile method allows both a graphical and a quantitative assessment of the prediction improvement caused by adding a new predictor to the reference model. The U-smile plot is easy and intuitive to interpret, with the largest smiles indicating the greatest prediction improvement over the reference model.

So far, we have observed that the U-smile method works for balanced or nearly balanced data, separates informative from non-informative parameters, and is reproducible. It seems to be robust to moderate multicollinearity between parameters included in the models. This method fulfils criteria for test propriety and allows efficient comparison of multiple candidate predictors.

The *BA* and *RB* coefficients are stratified by binary outcome class and measure the size of the prediction improvement. Thus, they offer a more granular view of the effect of the new marker compared to the Δ*AUC* of the *ROC* curves or the *I*. Using the U-smile method adds practical relevance and can lead to more informed decisions regarding variable selection.

## Supporting information

S1 Fig The prediction improvement-worsening (*PIW*) plots for each new model derived from the test dataset under the independent scenario—For the corresponding analysis as in Fig 7. (TIF)

S2 Fig The *ROC* curves of the reference and new models (as in Fig 5) derived from the test dataset under the independent scenario. (TIF)

S3 Fig The U-smile plots of the *RB* and *I* coefficients for each new model derived from the training and test datasets under the dependent scenario. (TIF)

S1 Appendix Additional calculations and tables. The relationship between the *BA* and *RB* coefficients and the Brier score. Tables with the values of the *RB* and *I* coefficients.
(ZIP)
