# Supplementary figures and images for: Visualising and quantifying the usefulness of new predictors stratified by outcome class: The U-smile method
Source: PLoS One. 2024 May 20;19(5):e0303276. doi: 10.1371/journal.pone.0303276 (PMC11104627; doi:10.1371/journal.pone.0303276)

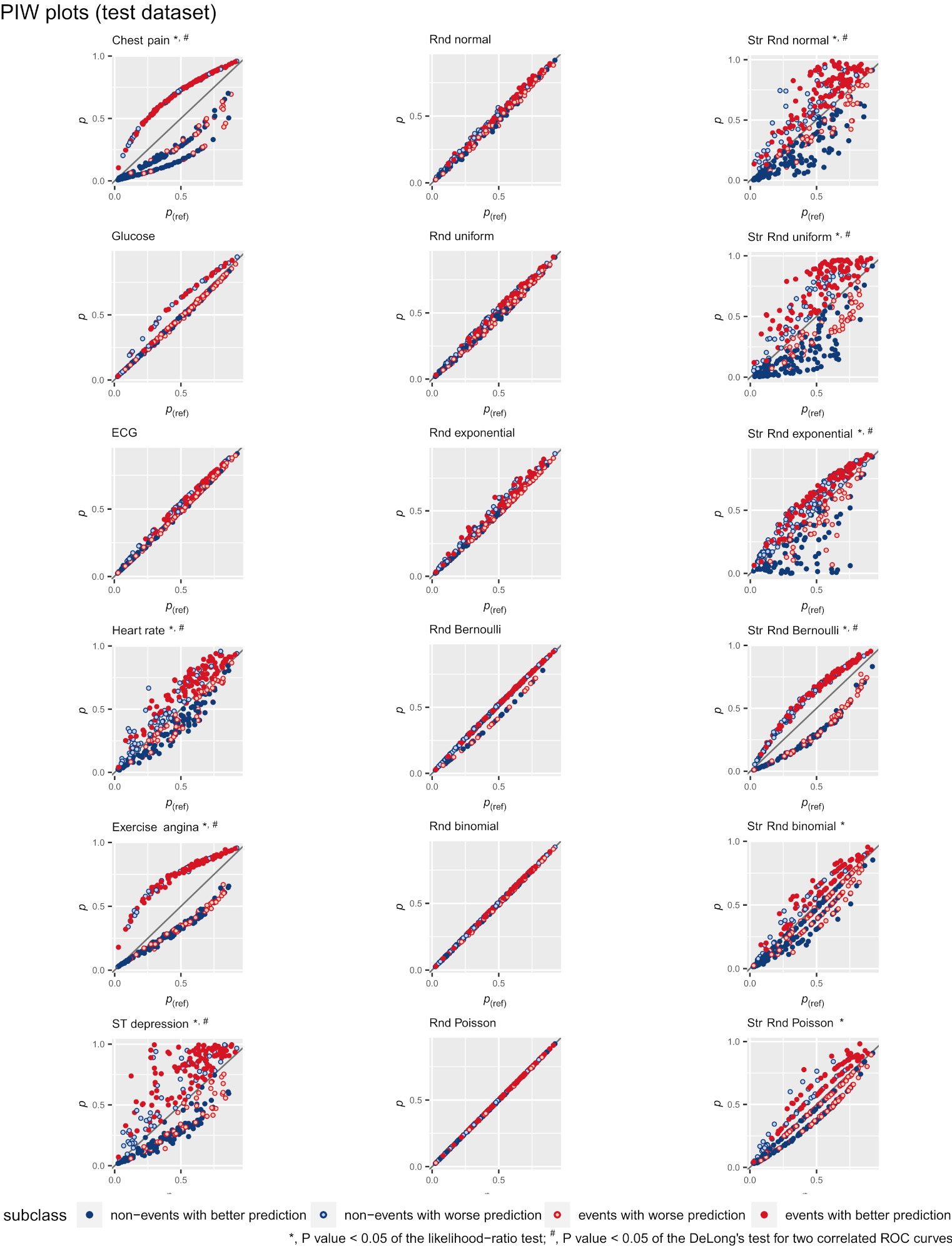

Supplement: S1 Fig — (TIF) [file pone.0303276.s001.tif]

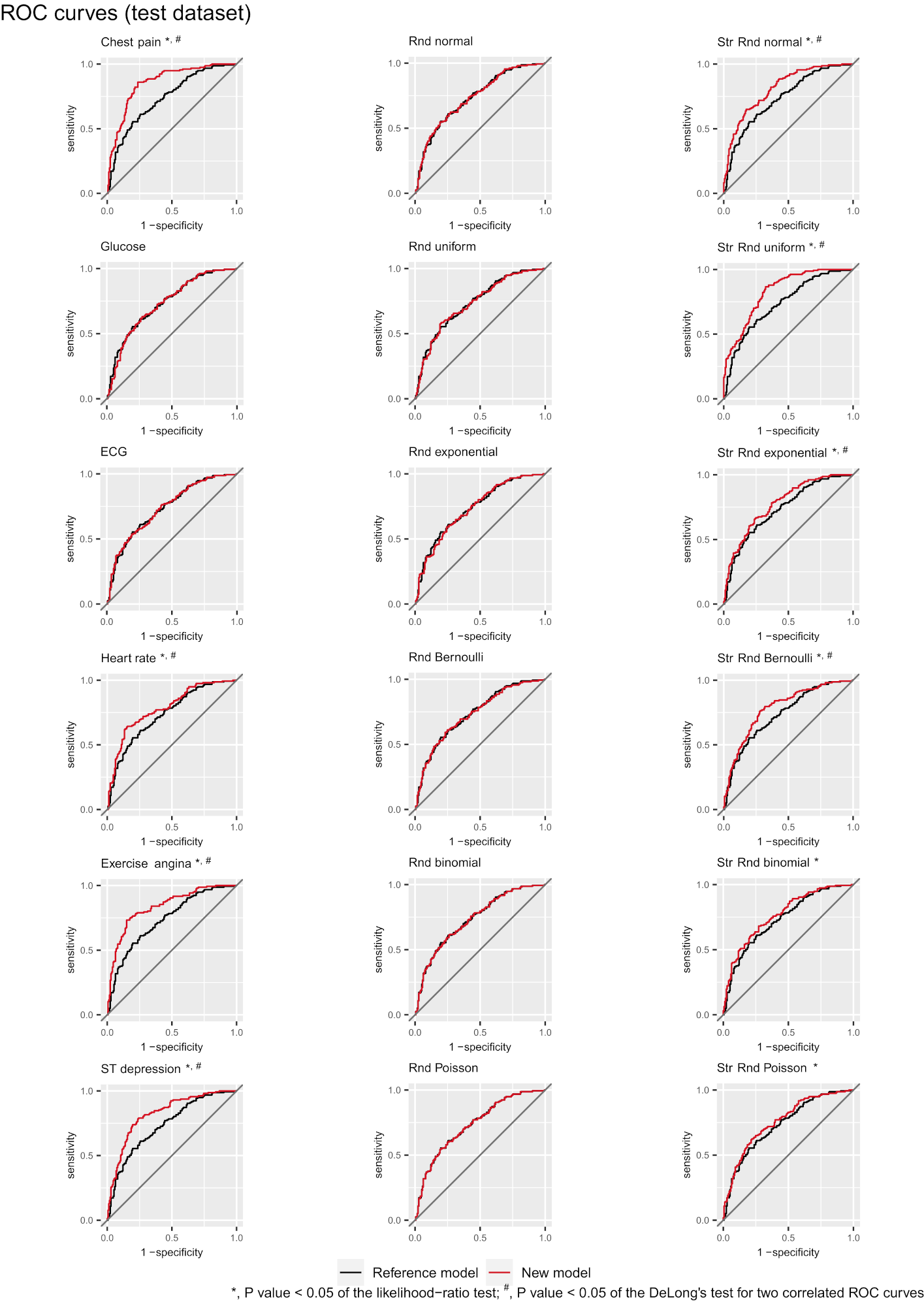

Supplement: S2 Fig — (TIF) [file pone.0303276.s002.tif]

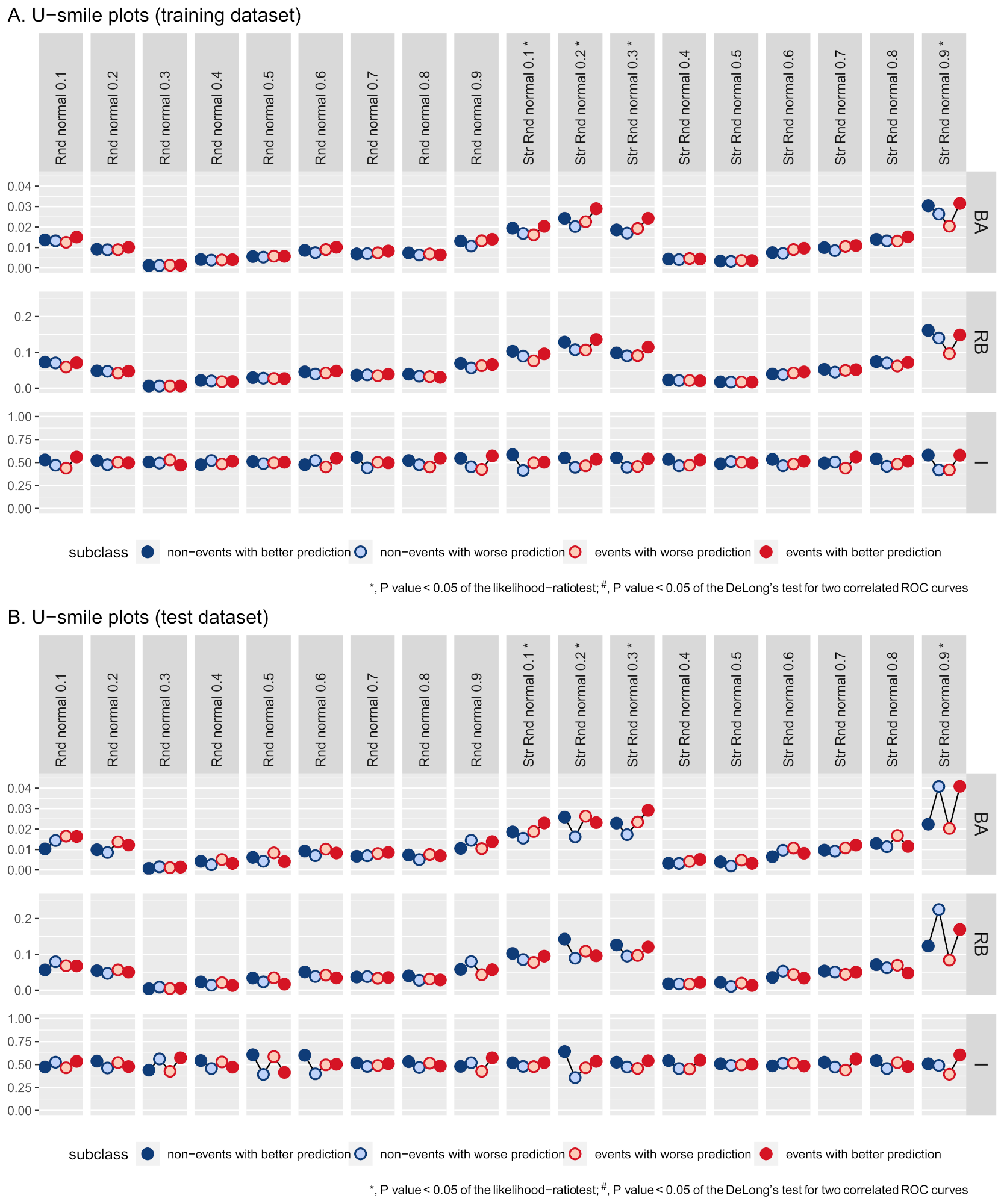

Supplement: S3 Fig — (TIF) [file pone.0303276.s003.tif]
