# Supplementary material for: Visualising and quantifying the usefulness of new predictors stratified by outcome class: The U-smile method
Source: PLoS One. 2024 May 20;19(5):e0303276. doi: 10.1371/journal.pone.0303276 (PMC11104627; doi:10.1371/journal.pone.0303276)
Supplement: S1 Appendix — The relationship between the BA and RB coefficients and the Brier score. Tables with the values of the RB and I coefficients. (ZIP) [file pone.0303276.s004.zip › S1_Appendix.pdf]

Additional calculations and tables

The $\boldsymbol{BA}$ and $\boldsymbol{RB}$ coefficients and the Brier score

Subscripts $_0$ and $_1$ denote the non-events and events, respectively, while superscripts $^-$ and $^+$ denote the worse and better prediction, respectively. The net BA coefficients and the net RB coefficients are defined in the main text.

If we define $w_{n0} = \frac{n_0}{n}$ and $w_{n1} = \frac{n_1}{n}$ as weights, the connection between the net BA coefficients and the Brier score is as follows:

$$\begin{aligned} w_{n0} \cdot BA_0 + w_{n1} \cdot BA_1 &= \frac{n_0}{n} \cdot \frac{\Delta SS_0^+ - \Delta SS_0^-}{n_0} + \frac{n_1}{n} \cdot \frac{\Delta SS_1^+ - \Delta SS_1^-}{n_1} \\ &= \frac{\Delta SS_0^+ - \Delta SS_0^- + \Delta SS_1^+ - \Delta SS_1^-}{n} \\ &= \frac{\Delta SS}{n} = \frac{SS_{(ref)}}{n} - \frac{SS}{n} = BS_{(ref)} - BS = \Delta BS, \end{aligned} \tag{1}$$

where ΔBS is the difference between the Brier score of the reference model, $BS_{(ref)} = \frac{SS_{(ref)}}{n}$, and the Brier score of the new model, $BS = \frac{SS}{n}$. Residual sum of squares, SS, is defined in the main text, where ΔSS is defined by the equation: $\Delta SS = \Delta SS_0^+ - \Delta SS_0^- + \Delta SS_1^+ - \Delta SS_1^- = SS_{(ref)} - SS$.

If we define $w_{s0} = \frac{SS_{0(ref)}}{SS_{(ref)}}$ and $w_{s1} = \frac{SS_{1(ref)}}{SS_{(ref)}}$ as weights, the connection between the net RB coefficients and the Brier score, $BSS = \frac{\Delta BS}{BS_{(ref)}} = 1 - \frac{BS}{BS_{(ref)}}$ is as follows:

$$\begin{aligned} w_{s0} \cdot RB_0 + w_{s1} \cdot RB_1 &= \frac{SS_{0(ref)}}{SS_{(ref)}} \cdot \frac{\Delta SS_0^+ - \Delta SS_0^-}{SS_{0(ref)}} + \frac{SS_{1(ref)}}{SS_{(ref)}} \cdot \frac{\Delta SS_1^+ - \Delta SS_1^-}{SS_{1(ref)}} \\ &= \frac{\Delta SS_0^+ - \Delta SS_0^- + \Delta SS_1^+ - \Delta SS_1^-}{SS_{(ref)}} \\ &= \frac{\Delta SS}{SS_{(ref)}} = \frac{SS_{(ref)} - SS}{SS_{(ref)}} \\ &= 1 - \frac{\frac{SS}{n}}{\frac{SS_{(ref)}}{n}} \\ &= 1 - \frac{BS}{BS_{(ref)}} = BSS, \end{aligned} \tag{2}$$

Values of the $\boldsymbol{RB}$ and $\boldsymbol{I}$ coefficients

Table 1. Values of the BA, RB and I coefficients stratified by outcome subclass for 18 new models derived from the training dataset under the independent scenario.

	Training dataset											
New model	BA_0^+	BA_0^-	BA_1^-	BA_1^+	RB_0^+	RB_0^-	RB_1^-	RB_1^+	I_0^+	I_0^-	I_1^-	I_1^+
**Real predictors**												
Chest pain	0.094	0.056	0.049	0.105	0.500	0.296	0.231	0.493	0.730	0.270	0.204	0.796
Glucose	0.012	0.010	0.012	0.012	0.065	0.051	0.056	0.058	0.753	0.247	0.726	0.274
ECG	0.008	0.007	0.008	0.008	0.040	0.038	0.036	0.039	0.684	0.316	0.624	0.375
Heart rate	0.043	0.030	0.029	0.042	0.227	0.158	0.137	0.198	0.626	0.374	0.420	0.580
Exercise angina	0.079	0.041	0.058	0.081	0.420	0.219	0.272	0.382	0.851	0.149	0.376	0.624
ST depression	0.083	0.030	0.057	0.085	0.442	0.160	0.268	0.401	0.805	0.195	0.350	0.650
**Random variables**												
Rnd normal	0.008	0.007	0.008	0.009	0.041	0.039	0.039	0.040	0.483	0.517	0.452	0.548
Rnd uniform	0.009	0.008	0.010	0.010	0.048	0.043	0.047	0.049	0.546	0.454	0.459	0.541
Rnd exponential	0.009	0.008	0.010	0.010	0.046	0.042	0.046	0.047	0.638	0.362	0.631	0.369
Rnd Bernoulli	0.010	0.010	0.010	0.011	0.055	0.054	0.049	0.054	0.224	0.776	0.172	0.828
Rnd binomial	0.002	0.002	0.002	0.003	0.012	0.012	0.012	0.013	0.379	0.621	0.363	0.637
Rnd Poisson	0.001	0.001	0.001	0.001	0.004	0.004	0.004	0.004	0.466	0.534	0.516	0.484
**Stratified random variables**												
Str Rnd normal	0.071	0.036	0.041	0.082	0.377	0.191	0.192	0.387	0.701	0.299	0.293	0.707
Str Rnd uniform	0.086	0.037	0.048	0.094	0.456	0.197	0.226	0.445	0.678	0.322	0.299	0.701
Str Rnd exponential	0.055	0.038	0.028	0.055	0.292	0.200	0.134	0.259	0.489	0.511	0.236	0.764
Str Rnd Bernoulli	0.068	0.048	0.045	0.073	0.362	0.253	0.211	0.342	0.592	0.408	0.242	0.758
Str Rnd binomial	0.034	0.025	0.029	0.038	0.179	0.135	0.135	0.179	0.655	0.345	0.452	0.548
Str Rnd Poisson	0.035	0.022	0.028	0.031	0.186	0.117	0.131	0.146	0.718	0.282	0.548	0.452

ECG, resting electrocardiographic changes

Table 2. Values of the BA, RB and I coefficients stratified by outcome subclass for 18 new models derived from the test dataset under the independent scenario.

	Test dataset											
New model	BA_0^+	BA_0^-	BA_1^-	BA_1^+	RB_0^+	RB_0^-	RB_1^-	RB_1^+	I_0^+	I_0^-	I_1^-	I_1^+
**Real predictors**												
Chest pain	0.095	0.046	0.054	0.117	0.524	0.255	0.225	0.483	0.763	0.237	0.229	0.771
Glucose	0.011	0.014	0.014	0.013	0.060	0.076	0.056	0.055	0.734	0.266	0.803	0.197
ECG	0.008	0.006	0.009	0.008	0.044	0.034	0.035	0.034	0.711	0.289	0.640	0.363
Exercise angina	0.078	0.037	0.056	0.109	0.433	0.205	0.233	0.453	0.844	0.156	0.357	0.643
ST depression	0.072	0.047	0.054	0.104	0.398	0.258	0.222	0.429	0.717	0.283	0.318	0.682
**Random variables**												
Rnd normal	0.007	0.006	0.007	0.008	0.037	0.031	0.031	0.034	0.503	0.497	0.459	0.541
Rnd uniform	0.011	0.008	0.013	0.008	0.059	0.045	0.054	0.031	0.590	0.410	0.586	0.414
Rnd exponential	0.007	0.009	0.010	0.011	0.037	0.049	0.040	0.045	0.590	0.410	0.605	0.395
Rnd Bernoulli	0.007	0.010	0.011	0.012	0.036	0.055	0.045	0.048	0.133	0.867	0.153	0.847
Rnd binomial	0.002	0.002	0.002	0.003	0.012	0.012	0.010	0.011	0.382	0.618	0.338	0.662
Rnd Poisson	0.001	0.001	0.001	0.001	0.004	0.004	0.004	0.004	0.416	0.584	0.510	0.490
**Stratified random variables**												
Str Rnd normal	0.061	0.049	0.027	0.081	0.336	0.269	0.112	0.338	0.682	0.318	0.280	0.720
Str Rnd uniform	0.074	0.045	0.051	0.096	0.409	0.248	0.211	0.398	0.717	0.283	0.331	0.669
Str Rnd exponential	0.045	0.042	0.031	0.062	0.251	0.231	0.129	0.257	0.451	0.549	0.236	0.764
Str Rnd Bernoulli	0.066	0.043	0.056	0.072	0.366	0.238	0.233	0.300	0.590	0.410	0.312	0.688
Str Rnd binomial	0.040	0.014	0.036	0.034	0.219	0.075	0.147	0.139	0.798	0.202	0.529	0.471
Str Rnd Poisson	0.037	0.022	0.030	0.034	0.204	0.120	0.125	0.143	0.763	0.237	0.573	0.427

ECG, resting electrocardiographic changes
